# Developmental stress predicts social network position

**DOI:** 10.1098/rsbl.2014.0561

**Published:** 2014-10

**Authors:** Neeltje J. Boogert, Damien R. Farine, Karen A. Spencer

**Affiliations:** 1 School of Psychology and Neuroscience, University of St. Andrews, St. Andrews KY16 9JP, UK; 2 Edward Grey Institute of Field Ornithology, University of Oxford, Oxford OX1 3PS, UK; 3 Department of Anthropology, University of California, Davis, CA 95616, USA; 4 Smithsonian Tropical Research Institute, Ancon, Panama

**Keywords:** corticosterone, development, foraging, social network, stress, zebra finch

## Abstract

The quantity and quality of social relationships, as captured by social network analysis, can have major fitness consequences. Various studies have shown that individual differences in social behaviour can be due to variation in exposure to developmental stress. However, whether these developmental differences translate to consistent differences in social network position is not known. We experimentally increased levels of the avian stress hormone corticosterone (CORT) in nestling zebra finches in a fully balanced design. Upon reaching nutritional independence, we released chicks and their families into two free-flying rooms, where we measured daily social networks over five weeks using passive integrated transponder tags. Developmental stress had a significant effect on social behaviour: despite having similar foraging patterns, CORT chicks had weaker associations to their parents than control chicks. Instead, CORT chicks foraged with a greater number of flock mates and were less choosy with whom they foraged, resulting in more central network positions. These findings highlight the importance of taking developmental history into account to understand the drivers of social organization in gregarious species.

## 1. Introduction

Social network position is increasingly being linked to fitness. The patterns of interactions between individuals form a social structure that can be captured using network analysis. Where individuals fit in this intricate social web can affect multiple aspects of life history in wild animal populations, including breeding success [1], the discovery of food resources [2], the diffusion of novel behaviours [3] and the transfer of pathogens [4]. However, despite considerable research using social networks to gain insight into population structure and processes, little is known about mechanisms that underlie inter-individual differences in network position and the stability of social relationships.

The quality of the developmental environment that an individual experiences may play an important role in determining its social behaviour in later life. Exposure to ‘stressful’ conditions in early development, such as intense sibling competition in large broods or food deprivation, can significantly increase endogenous levels of glucocorticoid hormones [5]. Experimental exposure to such stressful developmental conditions [6], or to elevated glucocorticoid hormone levels [7,8], has been shown to affect behaviours key to social interactions, such as competitive ability [7], mate choice [6] and social information use [8]. Individual variation in interaction rules can subsequently lead to different network positions, due to differences in attraction to conspecifics [9] or in the temporal maintenance of relationships [10].

In this study, we tested how experimentally-increased exposure to the avian glucocorticoid/‘stress’ hormone corticosterone (CORT) during early post natal development affected the social behaviour, and resulting network positions, of juvenile zebra finches (*Taeniopygia guttata*) once they reached nutritional independence. Although we did not directly measure CORT treatment-induced changes in the physiological stress response, the CORT treatment we used is known to elevate peak CORT concentrations and prolong CORT exposure in response to acute stressors in zebra finches [11] (although see [12]). In great tits (*Parus major*), individuals with higher peak CORT levels in response to acute stress tend to explore novel environments more slowly [13]. In the wild, slow explorers tend to stay in the same foraging flocks [14] and form few but stable associations, resulting in marginal network positions [10]. Based on these studies, we predicted that CORT-treated zebra finch chicks should maintain fewer and more stable social affiliations, resulting in less central positions (less well connected) than their control siblings.

## 2. Material and methods

Details of the housing and breeding conditions are provided in the electronic supplementary material.

### (a) Hormone treatment

Following [11], we started hormone treatments when chicks were 12 days old, assigning half of each of 13 broods to the CORT treatment (*n* = 20 chicks) and half to the control treatment (*n* = 17 chicks). Between post-hatching days 12 and 28 (when chicks become nutritionally independent), we fed CORT chicks 20 µl of CORT (Sigma Aldrich; 0.155 mg ml^−1^ in peanut oil) twice daily, giving a total dose of 6.2 µg CORT/day. This dose results in plasma CORT levels comparable to those naturally induced in chicks following exposure to an acute stressor (a standardized capture–handling–restraint protocol [15]), and it causes a prolonged CORT release following acute stress when measured 60 days post-hatching [11]. Control chicks were fed 20 µl of peanut oil when their siblings received CORT.

### (b) Social networks

When chicks were 37 ± 1 days old, they and their parents were fitted with passive integrated transponder (PIT) tags and, keeping families together, released on the same day into two identical free-flying rooms (3 × 3.1 × 3.2 m, containing seven and six families; electronic supplementary material, figure S1). Each room was equipped with two identical transparent feeders (28 × 28 × 10 cm) that contained finch seed at all times and were the only food sources available. Feeders were fitted with two radio-frequency identification antennae (Dorset ID) to record PIT tag codes as zebra finches freely entered and exited. After 5 days of habituation, we collected 35 days of PIT tag data between 27 February and 4 April 2013.

### (c) Statistical analyses

Analyses of differences in chick body mass due to CORT treatment are reported in the electronic supplementary material.

We inferred co-feeding events using a Gaussian Mixture Model. This algorithm identifies clusters of observations/PIT tag reads in non-uniform (or ‘bursty’) data streams [16], and circumvents subjective predefined thresholds (e.g. time windows) to detect flocks. We used the R package *asnipe* [17] to generate one network, based on the entire study period, and 35 daily networks, combining data from both rooms. Associations between individuals, or ‘edges', were calculated using the simple ratio index. An edge represents the probability of co-observing two individuals in the same versus different foraging groups (see the electronic supplementary material for further information, including repeatability analyses of individuals' network positions across days).

To determine whether our network captured the social structure in each room, we compared the strength of associations (edge weights) between (i) mated adults and non-paired adults, and (ii) family members and non-familial associations in the network, using weighted assortment coefficients (following [18]).

To investigate the effects of CORT treatment on network position, we used three centrality measures previously shown to predict important population dynamics in other species: (i) degree: the number and strength of associations [4], (ii) betweenness: the number of shortest paths that go through the focal individual [2,19] and (iii) eigenvector centrality: connectedness to other well-connected individuals [2]. Degree reflects gregariousness. Weighted degree is proportional to the mean group size individuals are found in, while unweighted degree is the sum of all associates over time. Degree has been shown to predict the spread of disease [4]. Betweenness captures the tendency of individuals to switch between different groups (high betweenness) versus staying with the same group (low betweenness). This mobility in social space can improve an individual's reproductive chances [19]. Eigenvector centrality captures the gregariousness of the focal individual's associates, and high values have been shown to result in earlier access to information about food sources [2]. We also investigated social differentiation (the coefficient of variation of edge weights) to determine whether differences in centrality arose because CORT birds associated more/less equally with conspecifics than control birds.

We used generalized linear mixed models to investigate the effects of CORT treatment on network position. Each model contained treatment, observation day, sex and interactions between treatment and day, and treatment and sex as fixed effects. Random effects were individual identity, nested within family and nested within test room.

In all statistical tests, we measured significance by comparing the coefficients of the models fitted to the observed data with coefficients calculated on 1000 permutations of the network (details in the electronic supplementary material).

## 3. Results

**Figure 1.**
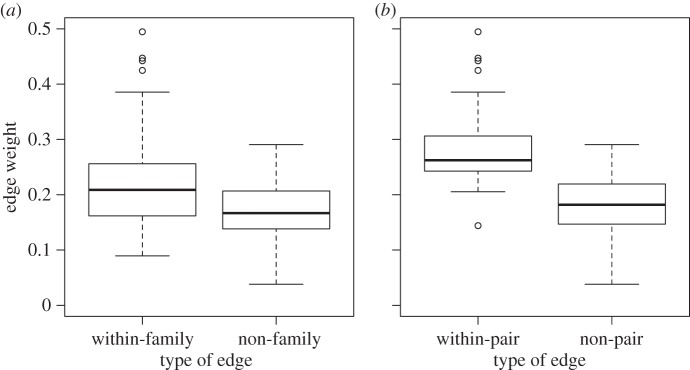
Edges between family members (*a*), and particularly between paired mates (*b*) were the strongest in the network. Box plots show the median and 25th and 75th percentiles, whiskers indicate values within 1.5 times the interquartile range and circles are outliers.

The overall network generated using PIT tag feeder visit data captured strong familial structure in each flock: within-family edges were significantly stronger than between-family edges (positive assortment within family: *r* ± s.e. = 0.091 ± 0.007, *p* < 0.001; figure 1*a*) and within-pair edges were significantly stronger than non-pair edges (positive pair assortment: *r* ± s.e. = 0.111 ± 0.027, *p* = 0.009; figure 1*b*).

**Figure 2.**
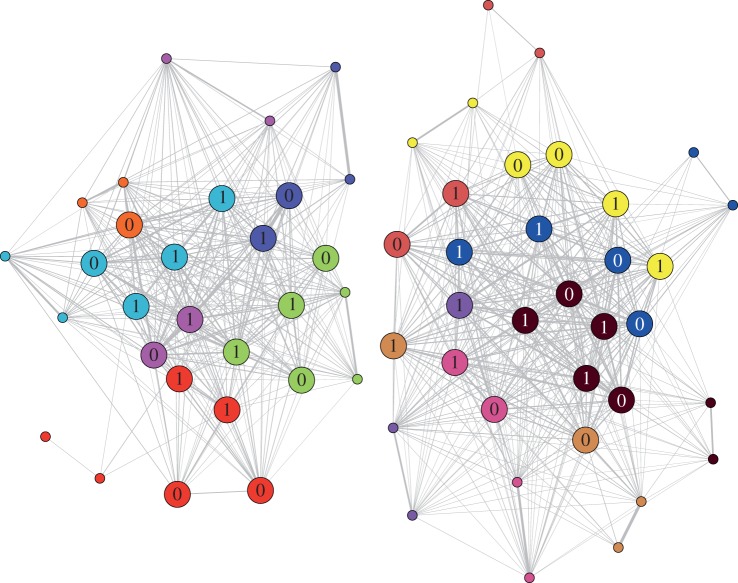
The social network represents the two zebra finch flocks in separate rooms. Birds were most strongly associated (thicker lines) with relatives (families are colour-coded), and the strongest foraging associations occurred between mated adults (small circles). Chicks (large circles) treated with CORT (1) associated less strongly with their parents and occupied more central positions in the network than control chicks (0).

CORT-treated birds did not differ from control birds in the size or number of foraging groups joined, nor in the number of feeder visits within each foraging bout (electronic supplementary material, figure S2). Thus, there was no difference in the weighted degree between treated and control birds (electronic supplementary material, table S1). However, CORT birds had a larger unweighted degree, i.e. they foraged with significantly more conspecifics each day (slope ± s.e. = 0.131 ± 0.067, *t* = 1.689, *p* = 0.012; electronic supplementary material, table S2). This resulted in lower social differentiation (i.e. CORT birds were less ‘choosy’ with whom they foraged; coefficient ± s.e. = −1.394 ± 1.033, *t* = −1.349, *p* < 0.001; electronic supplementary material, table S3) and greater betweenness (unweighted: coefficient ± s.e. = 0.099 ± 0.062, *t* = 1.603, *p* = 0.001, electronic supplementary material, table S4; weighted: electronic supplementary material, table S5). Thus, they occupied more central positions in the social networks than control birds (figure 2). CORT birds also associated less strongly with their parents throughout the study than control birds (coefficient ± s.e. = −0.008 ± 0.008, *t* = −0.861, *p* < 0.001; electronic supplementary material, table S9). However, CORT treatment did not affect eigenvector centrality (electronic supplementary material, tables S6–S8).

## 4. Discussion

Our study experimentally links developmental conditions with social network position. Zebra finches exposed to increased stress hormone levels during development (CORT chicks) foraged with more flock mates and more independently from their parents than control chicks, who were more ‘choosy’ with whom they foraged. This resulted in CORT chicks occupying consistently more central network positions than control chicks, by providing more ‘shortest paths’ between other birds (higher betweenness).

In great tits, central network positions are occupied by fast explorers [10] who tend to show lower peak CORT concentrations in response to a standardized stressor than slow explorers [13]. Interestingly, the opposite CORT–exploration relationship might hold true in zebra finches: fast explorers show *higher* peak CORT concentrations in response to stress than slow explorers. However, this correlation was only significant in a breeding line selected for low peak CORT responses to a standardized stressor, and not in the control or high peak CORT-selected lines [20]. If robust, this difference in the peak CORT–exploration relationship suggests that explorativeness could be the behavioural mediator of developmental stress-induced changes in social network positions. Developmental CORT exposure may have species-specific effects on exploration behaviour, possibly by changing feedback mechanisms in the hypothalamic–pituitary–adrenal axis [11,21]. Mesotocin, the avian homologue of the social bonding hormone oxytocin, may also play a role, as it modulates the physiological stress response [22] and promotes zebra finch flocking behaviour [23]. Finally, CORT-treated birds’ phenotypes may have changed such that flock mates treated them differently, which in turn could have caused the observed patterns. We are currently investigating whether these and/or other mechanisms underlie the observed link between CORT treatment and network position.

The clear familial and pair bond structures in our network demonstrate that inference from PIT tag records can capture the social structure of a study population, even in a captive setting. Admittedly, our captive flocks were relatively small and confined to remain together, potentially limiting the variance of individual network positions. This could explain why we did not find an effect of CORT treatment on eigenvector centrality (see the electronic supplementary material, tables S6–S8). Future research should replicate our study in wild bird populations using both CORT manipulations and naturally occurring stressors (e.g. sibling competition and food deprivation) and investigate which benefits and costs are associated with developmentally stressed birds' more central network positions in adolescence. Our results predict that developmental stress may expedite independence from parents. This may facilitate early dispersal from poor natal sites, such as those with high predation or low food availability, [24] or facilitate rapid food discovery [2].

Our study provides rare experimental insight into a mechanism underlying consistent differences in social network position. Developmental stress resulted in more independence and greater gregariousness, facilitating higher network centrality. Our findings suggest that taking developmental history into account will improve our understanding of the drivers of social organization in gregarious species.

## Supplementary Material

Boogert et al. Stress and Social Networks. Electronic Supplementary Material
