# Supplementary material for: Developmental stress predicts social network position
Source: Biol Lett. 2014 Oct;10(10):20140561. doi: 10.1098/rsbl.2014.0561 (PMC4272205; doi:10.1098/rsbl.2014.0561)
Supplement: Boogert et al. Stress and Social Networks. Electronic Supplementary Material [file rsbl20140561supp1.docx]

ELECTRONIC SUPPLEMENTARY MATERIAL

1. METHODS

(a) Housing and breeding conditions

We housed 24 adult zebra finch pairs in a single room containing wrought iron cages (50x50x50 cm) organized in two tiers stacked on top of each other. Each cage contained corn stalk pellets and hay bedding, a nest box, two perches, a cuttlefish bone, crushed oyster shells, a seed hopper and a water hopper, as well as an open water bowl and an open seed bowl. The water was supplemented with Johnsons Vitamin Drops for cage birds, and food containers were filled with mixed finch seed at all times. We also provided each pair daily with 1 tablespoon of Haith’s Egg Biscuit Food and with fresh spinach at least once a week. Birds were maintained at 20 ± 1°C ambient temperature on a 14:10 h light:dark cycle (lights on at 0700, off at 2100 hours), and lights were full-spectrum daylight. All birds could hear each other. We positioned opaque partitions in between adjacent cages to prevent immediate neighbours from disrupting each other’s breeding attempts (Felicity Muth, pers. comm.). Except for immediate neighbours, all birds could see each other.

We checked all breeding cages daily and recorded the laying date of each egg. Upon being laid, we replaced each egg with a plastic finch dummy egg to synchronize hatching dates to standardize the timing of the stress hormone treatment across the brood. When no more eggs were laid on two subsequent days, we considered the clutch finished and replaced all dummy eggs with the pair’s real eggs. We recorded each chick’s hatching date and painted its toe nails and head fluff with a unique colour nail varnish to distinguish it from its brood mates. We weighed chicks on post-hatching days 5, 12, 20 and 28. As chicks with the same hatching date varied in body mass, we counterbalanced CORT and control treatments across higher and lower body mass within each brood.

(b) Free-flying rooms

Figure S1 shows a diagram (not drawn to scale) of the free-flying rooms. We positioned opaque white corrugated plastic partitions next to the feeders such that while in/around one feeder, birds could not see who were present in/around the other feeder. To provide ample perching space, we also fitted each room with eucalyptus branches and attached dowels and ropes 2 m from ground level across the room (not shown). Each room also contained 3 water dishes that served as bird baths (30 cm diameter).


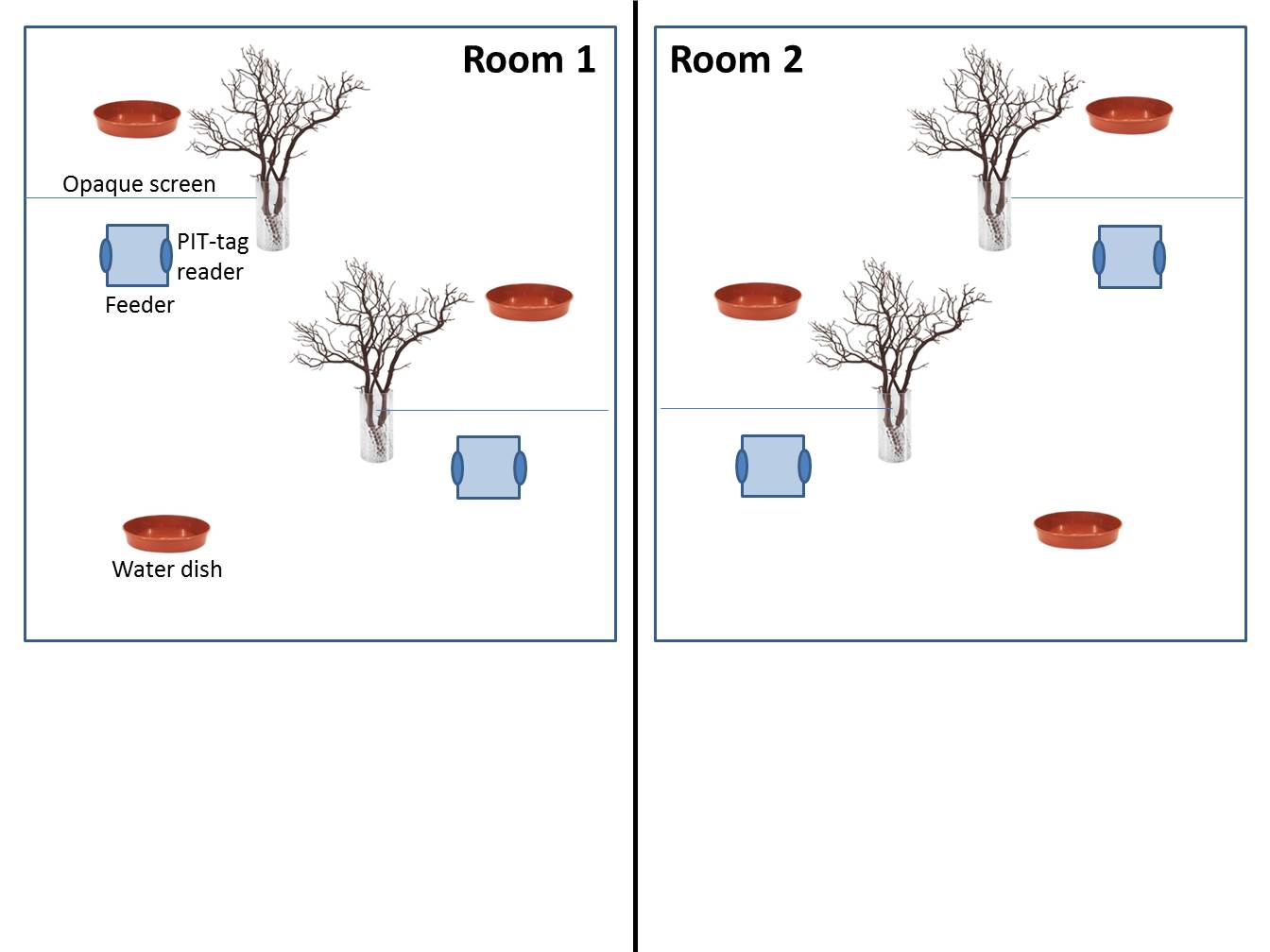


**Figure S1.** Test room set-up.

(c) Study timeline

| **Date** | **Event** |
| --- | --- |
| 23/12/2012 | Pairs put in breeding cages |
| 15/01/2013-20/01/2013 | Eggs hatch |
| 27/01/2013-17/02/2013 | CORT-treatment of chicks aged 12-28 days |
| 22/02/2013 | All birds fitted with PIT-tags and released into  2 free-flying rooms:  room 1: 7 families, room 2: 6 families |
| 22/02/2013-26/02/2013 | Habituation to free-flying rooms |
| 27/02/2013-04/04/2013 | Collection of 35 days of PIT tag data |

(d) Inferring social structure from foraging networks

We used the following methods to construct the social networks from foraging associations: (i) individuals were individually identified when they accessed the feeders by their unique (10-digit hexadecimal code) electronic PIT-tag attached to a plastic leg ring. (ii) Tags were recorded by RFID antennae integrated into the feeder entrances and the bird identity, feeder identity, time and date of each feeder visit were saved onto the internal memory of the PIT-tag reader devices (Dorset ID). (iii) An association was defined as two individuals co-occurring in the same social group at the same feeder, using a ‘gambit of the group’ approach [1]. (iv) Groups were defined as peaks in activity in the temporal data-stream at feeders, which were identified using a clustering algorithm (i.e. a Gaussian-mixed model [2]). The average time a flock spent at a feeder was 290 seconds, and flocks contained on average 7 individuals. While the co-occurrence of two individuals may not necessarily be meaningful for a single event, the social network links used in this study were built up from over 8000 group observations, representing a complete record of every single feeder visit of each individual over the 35 days. Further, all P- values were obtained by comparing the observed network to random networks generated from the same data stream. (v) Networks were weighted, with the strength of the link between nodes calculated as the total number of observations of each dyad observed foraging in the same flock, divided by the time those individuals were observed apart (the simple ratio index [3]). This process was applied both to the entire data set (all 8000 groups at once) to create a single network, and to daily subsets of the data to create daily networks for each of the two rooms. (vi) Foraging networks can be used to capture the social structure (preferred and avoided relationships) of bird communities as they represent repeated coordination in the behaviour of pairs of individuals. For example [4,5] identified the interaction rules used by wild tits (family *Paridae*) when choosing whether to forage in patches with conspecifics or not. These rules then scale up to the patterns observed in a winter-long population-wide social network [6]. In the present study, the networks are used to capture familial and pair-wise foraging bouts, and the presence of socially-differentiated (preferred or avoided) relationships within the aviary groups due to CORT-treatment.

(e) Statistical analyses

*Effect of CORT-treatment on zebra finch weight*

We used linear mixed effects model in R to test whether CORT-chicks differed in body weight from control chicks on the first (12 days post-hatching) and on the final day (28 days post-hatching) of CORT-treatment. Models included chick weight as the response variable, treatment as a fixed effect and as random effects the no. of fledglings nested within no. of eggs nested within family.

*Repeatability of social network position*

To measure the repeatability, or consistency, of individuals’ network positions across days, we calculated the intra-class correlation coefficient (following [8]) of individual degree. This coefficient is estimated by measuring the proportion of the variance in the model that is explained by individual identity (fitted as a random effect).

*Familial and pair-wise structures in the social network*

We used the assortativity coefficient to determine if associations were stronger in family units (assorted by family), using [7]. This was applied to the full network, where each family was assigned a unique family identifier, thus testing whether the edge weights within these family units were significantly stronger than those between individuals in different family units. To test for differences between- vs within-pair edges, we repeated this process on only the subset of individuals that were adults (i.e. the network containing only adults). The results of these analyses are reported in the main text.

*Using permutation tests to assess the significance of social network structures*

Because node-level observations in a network are non-independent, we calculated significance in all statistical tests (assortment, GLMMs and repeatability) by comparing the coefficients of the model (or the test statistic in the case of assortment) calculated using the observed data to the coefficients calculated using 1000 permutations of the network. Permutations were performed in *asnipe* following the algorithm first described in [9]. This method enabled us to maintain the number of observations of each individual by randomly swapping the observations of two individuals at each permutation step. Further, we restricted these swaps to occur between pairs of observations occurring at the same feeder (thus also in the same room) and on the same day. The resulting daily networks were recalculated at each step (each step contained 100 swaps) and the P-value was calculated by comparing the observed slope to the distribution of slope parameters estimated from each of the 1000 permuted networks.

The raw data can be downloaded from the Dryad repository (www.datadryad.org).

2. RESULTS

(a) Effect of CORT-treatment on zebra finch weight

Before the start of CORT-treatment, CORT-chicks and control chicks did not differ significantly in body weight (weight post-hatching day 5: difference±SE=-0.038±0.224, df=24, *t*=-0.168, *p*=0.868). As expected, by post-hatching day 20, after 8 days of CORT-treatment, CORT-chicks were significantly lighter than control chicks (difference±SE=-0.508±0.229, df=24, *t*=-2.217, *p*=0.036), and this effect was even more pronounced at the end of treatment, by post-hatching day 28 (difference±SE=-0.972±0.305, df=24, *t*=-3.188, *p*=0.004).

(b) Repeatability of social network position

Individual network degree across all birds was highly repeatable across days (R=0.77, 95%CI=0.67-0.84, *p*=0.030), suggesting that the behaviour of individuals was more consistent across the 35 daily networks than expected from the permutations. Further, we found no interaction between test day and treatment in any model, suggesting that behaviour did not become more strongly or weakly differentiated over time.

(c) Effect of CORT-treatment on general feeder visit patterns

CORT-treated birds did not differ from control birds in the size or number of foraging groups joined (group size: *t*=-0.184, df=32, *p*=0.855, difference=-0.055, 95%CI=-0.56-0.67; number of groups: *t*=-0.720, df=32, *p*=0.477, difference=46.0, 95%CI=-176-84), nor in the number of feeder visits in each group foraging bout (*t*=-0.471, df=32, *p*=0.640, difference=0.151, 95%CI=-0.80-0.50; see Figure S2). However, CORT-birds were connected to all of their roommates in over 95% of daily observations, whereas for non CORT-birds this was the case in only 85% of daily observations.


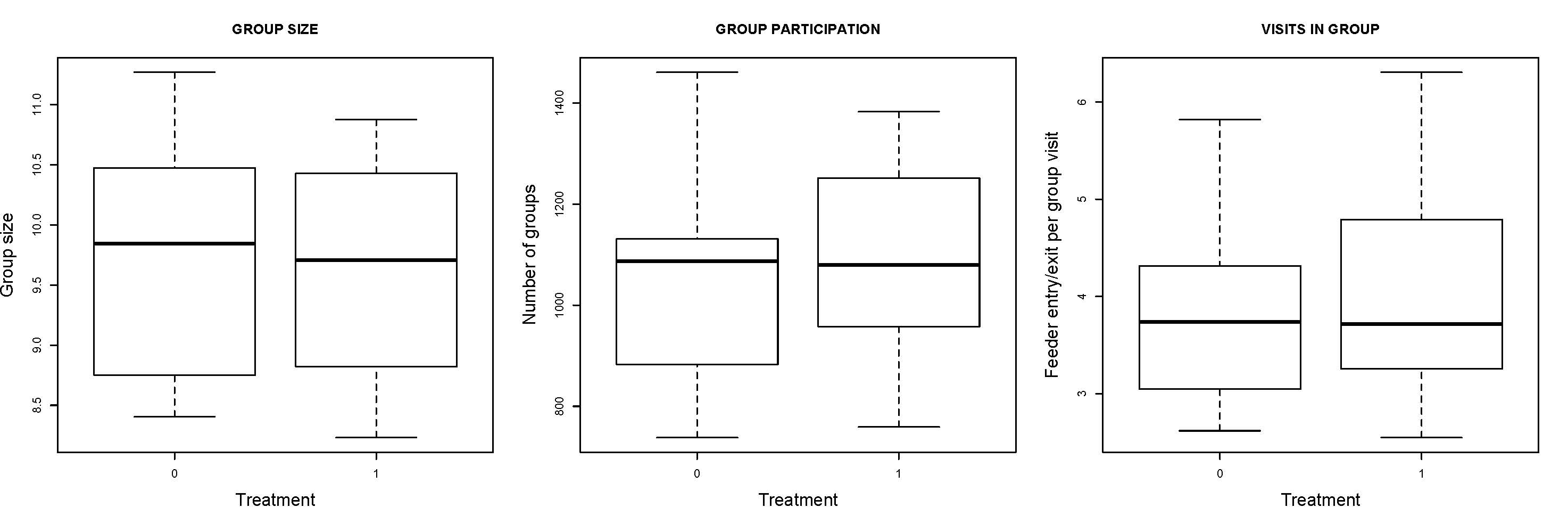


**Figure S2.** The effect of CORT-treatment on the size of the foraging groups joined, the number of foraging groups joined, and the number of visits to the feeder during each group-foraging bout. Differences between CORT-treated (1) and control chicks (0) were not significant. Box plots show the median and 25th and 75th percentiles and whiskers indicate the values within 1.5 times the interquartile range.

(d) Effect of CORT-treatment on social network structure

Tables S1-S7 contain the results of generalised linear mixed models (GLMMs) testing the effects of CORT-treatment on Degree, Social Differentiation, Betweenness, Eigenvector Centrality and Association strength to parents. Social differentiation is measured using the coefficient of variation (CV) of the association strengths of each individual [10]. This measure captures how equal each individual’s relationships are with others, where a high CV represents preferred and avoided relationships, and a low CV represents equal associations across all conspecifics in the same room. Each model contained treatment, observation day (1-35), sex, an interaction between treatment and day, and an interaction between treatment and sex, as fixed effects, and individual identity nested within family nested within room (1-2) as random effects. Significant fixed effects are highlighted in grey. For each response variable, results are shown for a) the full models (i.e. including all fixed effects), and, when Treatment was found to be significant, for b) the full models from which non-significant fixed effects (i.e. as apparent from the full-model-run) were removed. Note that Sex and Treatment*Sex are significant in some models, but the effect sizes of these predictors are always much smaller than the effect size of Treatment.

***Table S1*** *Effect of CORT-treatment on weighted Degree (mean group size): Full Model Results*

| **Fixed effects:** | **Beta** | **SE** | **T** | **P_rand_** |
| --- | --- | --- | --- | --- |
| Treatment | 0.023 | 0.222 | 0.102 | 0.045 |
| Day | 0.052 | 0.018 | 2.960 | 0.003 |
| Sex | -0.261 | 0.202 | -1.284 | 0.201 |
| Treatment*Sex | 0.347 | 0.295 | 1.179 | 0.197 |
| Treatment*Day | -0.006 | 0.006 | -1.140 | 0.859 |
|  | | | | |
| **Random effects:** | **Variance** | **SD** | **% total** |  |
| ID | 1.009 | 0.395 | 41% |  |
| Family | 0.000 | 0.000 | 0% |  |
| Room | 1.098 | 0.331 | 45% |  |

***Table S2.1*** *Effect of CORT-treatment on Degree (number of associates): Full Model Results*

| **Fixed effects:** | **Beta** | **SE** | **T** | **P_rand_** |
| --- | --- | --- | --- | --- |
| Treatment | 0.169 | 0.144 | 1.171 | 0.045 |
| Day | 0.015 | 0.014 | 1.056 | 0.593 |
| Sex | -0.182 | 0.097 | -1.858 | 0.599 |
| Treatment*Sex | 0.209 | 0.135 | 1.555 | 0.956 |
| Treatment*Day | -0.009 | 0.005 | -1.485 | 0.122 |
|  | | | | |
| **Random effects:** | **Variance** | **SD** | **% total** |  |
| ID | 1.009 | 0.395 | 41% |  |
| Family | 0.000 | 0.000 | 0% |  |
| Room | 1.098 | 0.331 | 45% |  |

***Table S2.2*** *Effect of CORT-treatment on Degree (number of associates): Model Results when non-significant Fixed Effects in Full Model are removed.*

| **Fixed effects:** | **Beta** | **SE** | **T** | **P_rand_** |
| --- | --- | --- | --- | --- |
| Treatment | 0.131 | 0.067 | 1.689 | 0.012 |
|  | | | | |
| **Random effects:** | **Variance** | **SD** | **% total** |  |
| ID | 0.632 | 0.178 | 22% |  |
| Family | 0.000 | 0.000 | 0% |  |
| Room | 1.109 | 0.972 | 38% |  |

***Table S3.1*** *Effect of CORT-treatment on social differentiation (CV): Full Model Results*

| **Fixed effects:** | **Beta** | **SE** | **T** | **P_rand_** |
| --- | --- | --- | --- | --- |
| Treatment | -2.548 | 1.259 | -2.023 | <0.001 |
| Day | -0.132 | 0.104 | -1.272 | 0.123 |
| Sex | -0.555 | 1.054 | -0.527 | <0.001 |
| Treatment*Sex | 0.860 | 1.499 | 0.574 | 0.041 |
| Treatment*Day | 0.064 | 0.040 | 1.604 | 0.213 |
|  | | | | |
| **Random effects:** | **Variance** | **SD** | **% total** |  |
| ID | 157.97 | 11.06 | 74% |  |
| Family | 0.70 | 0.88 | 0% |  |
| Room | 2.42 | 1.55 | 1% |  |

***Table S3.2*** *Effect of CORT-treatment on social differentiation (CV): Model Results when non-significant Fixed Effects in Full Model are removed.*

| **Fixed effects:** | **Beta** | **SE** | **T** | **P_rand_** |
| --- | --- | --- | --- | --- |
| Treatment | -1.394 | 1.033 | -1.349 | <0.001 |
| Sex | -0.555 | 1.054 | -0.527 | <0.001 |
| Treatment*Sex | 0.860 | 1.499 | 0.574 | <0.001 |
|  | | | | |
| **Random effects:** | **Variance** | **SD** | **% total** |  |
| ID | 156.93 | 11.45 | 74% |  |
| Family | 0.91 | 0.99 | 0% |  |
| Room | 2.34 | 1.53 | 1% |  |

***Table S4.1*** *Effect of CORT-treatment on unweighted Betweenness: Full Model Results*

| **Fixed effects:** | **Beta** | **SE** | **T** | **P_rand_** |
| --- | --- | --- | --- | --- |
| Treatment | 0.151 | 0.082 | 1.827 | <0.001 |
| Day | 0.125 | 0.142 | 0.898 | 0.598 |
| Sex | -0.138 | 0.091 | -1.512 | 0.856 |
| Treatment*Sex | 0.153 | 0.126 | 1.224 | 0.975 |
| Treatment*Day | -0.006 | 0.006 | -0.948 | 0.941 |
|  | | | | |
| **Random effects:** | **Variance** | **SD** | **% total** |  |
| ID | 0.000 | 0.000 | 0% |  |
| Family | 0.000 | 0.000 | 0% |  |
| Room | 0.023 | 0.152 | 3% |  |

***Table S4.2*** *Effect of CORT-treatment on unweighted Betweenness: Model Results when non-significant Fixed Effects in Full Model are removed.*

| **Fixed effects:** | **Beta** | **SE** | **T** | **P_rand_** |
| --- | --- | --- | --- | --- |
| Treatment | 0.099 | 0.062 | 1.603 | 0.001 |
|  | | | | |
| **Random effects:** | **Variance** | **SD** | **% total** |  |
| ID | 10.030 | 3.210 | 27% |  |
| Family | 0.000 | 0.000 | 0% |  |
| Room | 276.44 | 5.075 | 69% |  |

***Table S5.1*** *Effect of CORT-treatment on weighted Betweenness: Full Model Results*

| **Fixed effects:** | **Beta** | **SE** | **T** | **P_rand_** |
| --- | --- | --- | --- | --- |
| Treatment | 1.648 | 1.262 | 1.306 | <0.001 |
| Day | -0.065 | 0.045 | 1.451 | 1.000 |
| Sex | -0.256 | 0.960 | -0.267 | <0.001 |
| Treatment*Sex | 0.055 | 0.045 | 1.211 | 0.995 |
| Treatment*Day | 0.056 | 1.338 | 0.042 | 0.895 |
|  | | | | |
| **Random effects:** | **Variance** | **SD** | **% total** |  |
| ID | 4.835 | 3.020 | 7% |  |
| Family | 0.279 | 0.528 | <1% |  |
| Room | 0.000 | 0.000 | 0% |  |

***Table S5.2*** *Effect of CORT-treatment on weighted Betweenness: Model Results when non-significant Fixed Effects in Full Model are removed.*

| **Fixed effects:** | **Beta** | **SE** | **T** | **P_rand_** |
| --- | --- | --- | --- | --- |
| Treatment | 0.648 | 0.618 | 1.407 | <0.001 |
| Sex | -0.221 | 0.638 | -0.347 | <0.001 |
|  | | | | |
| **Random effects:** | **Variance** | **SD** | **% total** |  |
| ID | 4.737 | 2.996 | 7% |  |
| Family | 0.237 | 0.487 | <1% |  |
| Room | 0.000 | 0.000 | 0% |  |

***Table S6.*** *Effect of CORT-treatment on Eigenvector Centrality (unweighted network): Full Model Results*

| **Fixed effects:** | **Beta** | **SE** | **T** | **P_rand_** |
| --- | --- | --- | --- | --- |
| Treatment | 0.000 | 0.003 | 0.076 | 0.896 |
| Day | 0.000 | 0.000 | 0.172 | 0.576 |
| Sex | 0.000 | 0.002 | -0.066 | 0.742 |
| Treatment*Sex | 0.000 | 0.000 | 0.231 | 0.252 |
| Treatment*Day | 0.000 | 0.000 | -0.059 | 0.614 |
|  | | | | |
| **Random effects:** | **Variance** | **SD** | **% total** |  |
| ID | 0.000 | 0.000 | 0% |  |
| Family | 0.000 | 0.000 | 0% |  |
| Room | 0.013 | 0.115 | 59% |  |

***Table S7.*** *Effect of CORT-treatment on Eigenvector Centrality (weighted network): Full Model Results*

| **Fixed effects:** | **Beta** | **SE** | **T** | **P_rand_** |
| --- | --- | --- | --- | --- |
| Treatment | 0.004 | 0.008 | 0.468 | 0.550 |
| Day | 0.000 | 0.000 | 1.053 | 0.341 |
| Sex | -0.007 | 0.006 | -1.151 | <0.001 |
| Treatment*Sex | -0.001 | 0.001 | -0.335 | 0.002 |
| Treatment*Day | 0.008 | 0.008 | 0.963 | 0.566 |
|  | | | | |
| **Random effects:** | **Variance** | **SD** | **% total** |  |
| ID | 0.000 | 0.000 | 0% |  |
| Family | 0.000 | 0.000 | 0% |  |
| Room | 0.007 | 0.090 | 57% |  |

***Table S8.*** *Effect of CORT-treatment on Eigenvector Centrality (weighted thesholded network: weaker edges are removed to create a sparser network). These results suggest that CORT-treatment may have affected Eigenvector Centrality after all, when the network is less fully connected. However, significance is not tested as thresholding can lead to spurious p-values;* [7]*.*

| **Fixed effects:** | **Beta** | **SE** | **t** |
| --- | --- | --- | --- |
| Treatment | 0.235 | 0.194 | 1.210 |
| Day | 0.063 | 0.022 | 2.911 |
| Sex | -0.069 | 0.157 | -0.436 |
| Treatment*Sex | -0.002 | -0.002 | 0.488 |
| Treatment*Day | -0.007 | 0.007 | -1.028 |
|  | | | |
| **Random effects:** | **Variance** | **SD** | **% total** |
| ID | 0.145 | 0.381 | 4% |
| Family | 0.150 | 0.387 | 4% |
| Room | 0.352 | 0.593 | 9% |

***Table S9.1*** *Effect of CORT-treatment on the strength of association with parents: Full Model Results*

| **Fixed effects:** | **Beta** | **SE** | **T** | **P_rand_** |
| --- | --- | --- | --- | --- |
| Treatment | -0.047 | 0.011 | -3.568 | <0.001 |
| Day | 0.001 | 0.001 | 1.297 | 0.008 |
| Sex | 0.002 | 0.010 | 0.227 | 0.905 |
| Treatment*Sex | 0.002 | 0.002 | 0.104 | 0.002 |
| Treatment*Day | 0.001 | 0.001 | 1.019 | 0.170 |
|  | | | | |
| **Random effects:** | **Variance** | **SD** | **% total** |  |
| ID | 0.001 | 0.030 | 28% |  |
| Family | 0.000 | 0.000 | 0% |  |
| Room | 0.000 | 0.005 | <1% |  |

***Table S9.2*** *Effect of CORT-treatment on the strength of association with parents: Model Results when non-significant Fixed Effects in Full Model are removed.*

| **Fixed effects:** | **Beta** | **SE** | **T** | **P_rand_** |
| --- | --- | --- | --- | --- |
| Treatment | -0.008 | 0.008 | -0.861 | <0.001 |
| Day | 0.001 | 0.001 | 2.81 | 0.350 |
| Treatment*Sex | 0.016 | 0.013 | 1.200 | 0.723 |
|  | | | | |
| **Random effects:** | **Variance** | **SD** | **% total** |  |
| ID | 0.000 | 0.000 | 3% |  |
| Family | 0.000 | 0.000 | 0% |  |
| Room | 0.000 | 0.000 | 0% |  |

References

1. Croft, D. P., Madden, J. R., Franks, D. W. & James, R. 2011 Hypothesis testing in animal social networks. *Trends Ecol. Evol.* **26**, 502–7. (doi:10.1016/j.tree.2011.05.012)

2. Psorakis, I., Roberts, S. J., Rezek, I. & Sheldon, B. C. 2012 Inferring social network structure in ecological systems from spatio-temporal data streams. *J. R. Soc. Interface*, doi:10.1098/rsif.2012.0223.

3. Cairns, S. J. & Schwager, S. J. 1987 A comparison of association indexes. *Anim. Behav.* **35**, 1454–1469.

4. Aplin, L. M., Farine, D. R., Mann, R. P. & Sheldon, B. C. 2014 Individual-level personality influences social foraging and collective behaviour in wild birds. *Proc. R. Soc. B Biol. Sci.* **281**, 20141016.

5. Farine, D. R., Aplin, L. M., Garroway, C. J., Mann, R. P. & Sheldon, B. C. 2014 Collective decision-making and social interaction rules in mixed-species flocks of songbirds. *Anim. Behav.* **95**, 173-182.

6. Aplin, L. M., Farine, D. R., Morand-Ferron, J., Cole, E. F., Cockburn, A. & Sheldon, B. C. 2013 Individual personalities predict social behaviour in wild networks of great tits (*Parus major*). *Ecol. Lett.* **16**, 1365–72. (doi:10.1111/ele.12181)

7. Farine, D. R. 2014 Measuring phenotypic assortment in animal social networks: weighted associations are more robust than binary edges. *Anim. Behav.* **89**, 141–153. (doi:10.1016/j.anbehav.2014.01.001)

8. Nakagawa, S. & Schielzeth, H. 2010 Repeatability for Gaussian and non-Gaussian data: a practical guide for biologists. *Biol. Rev. Camb. Philos. Soc.* **85**, 935–56. (doi:10.1111/j.1469-185X.2010.00141.x)

9. Bejder, L., Fletcher, D. & Bräger, S. 1998 A method for testing association patterns of social animals. *Anim. Behav.* **56**, 719–725. (doi:10.1006/anbe.1998.0802)

10. Whitehead, H. 2008 *Analyzing animal societies: quantitative methods for vertebrate social analysis*. Chicago: University of Chicago Press.
